# The Impact of Leukemia Inhibitory Factor on *Sirtuin1*, *Ilt‐4*, and Related microRNAs Expression in a Mouse Model of Recurrent Pregnancy Loss

**DOI:** 10.1002/rmb2.70062

**Published:** 2026-06-18

**Authors:** Seyed Mohammad Seifati, Fateme Zare, Hossein Ansariniya, Farzaneh Fesahat, Samaneh Harimi, Maryam Imani, Hossein Hadinedoushan

**Affiliations:** ^1^ Reproductive Immunology Research Center Shahid Sadoughi University of Medical Sciences Yazd Iran; ^2^ Department of Immunology Shahid Sadoughi University of Medical Sciences Yazd Iran

**Keywords:** ILT‐4, leukemia inhibitory factor, microRNAs, recurrent pregnancy loss, sirtuin 1

## Abstract

**Purpose:**

Leukemia Inhibitory Factor (LIF) plays a crucial role in implantation and early pregnancy maintenance. However, its regulatory relationships with molecular markers such as Sirtuin 1 (SIRT1), immunoglobulin‐like transcript 4 (ILT‐4), and specific microRNAs (miRNA) in recurrent pregnancy loss (RPL) remain underexplored.

**Methods:**

This study investigates the effect of LIF on the expression of *Sirt1*, *Ilt‐4*, and related miRNAs using a mouse model of RPL. Female CBA/J mice mated with DBA/2 J males received LIF treatment. Gene and miRNA expression levels were assessed via quantitative PCR.

**Results:**

The LIF‐treated group showed significant upregulation of *Sirt1* on Days 7 and 14. *Ilt‐4* expression also increased significantly on Days 7 and 14 in the LIF group. Among miRNAs, *mir‐138‐5p* was significantly upregulated on Day 14 (*p* = 0.0001), while *mir‐223‐3p* was downregulated on Days 7 (*p* = 0.02) and 14 (*p* = 0.001). *mir‐199a‐5p* showed significant downregulation on Days 7 (*p* < 0.0001) and 14 (*p* = 0.0001). Additionally, *mir‐155‐5p* demonstrated a significant decrease in the LIF‐treated group compared to the PBS group (*p* < 0.0001).

**Conclusions:**

These results suggest that LIF modulates critical molecular pathways in RPL by influencing the expression of essential genes and miRNAs, highlighting its potential as a therapeutic target for pregnancy‐related complications.

## 1 Introduction

Recurrent pregnancy loss (RPL), or recurrent miscarriage, is a prevalent reproductive issue impacting about 5% of couples globally. The World Health Organization (WHO) defines it as the loss of three or more consecutive pregnancies before the 20th week of gestation [1]. Most researchers associate RPL with parental chromosomal abnormalities, uterine anomalies (congenital or acquired), endocrine disorders, immunological factors such as antiphospholipid syndrome, infections, and thrombophilia [2, 3].

Given the limitations of studying miscarriage in humans, mouse models provide a valuable alternative. There are several models of miscarriage, each triggered by different factors. However, the most extensively studied model is the CBA/J × DBA/2 J model. Mating DBA/2 J males with CBA/J females provides a homologous RPL model for researchers [4].

At the molecular level, leukemia inhibitory factor (LIF), a pleiotropic cytokine of the interleukin‐6 (IL‐6) family, plays a pivotal role in pregnancy establishment [5, 6]. LIF is highly expressed in uterine endometrial glands prior to blastocyst implantation in both mice and humans, influenced by rising steroid hormone levels during the menstrual cycle [7]. Reduced LIF expression has been observed in women with infertility, with rare LIF gene mutations reported in infertile women [8].

Another important regulator, immunoglobulin‐like transcript 4 (ILT‐4), is a member of the immunoglobulin superfamily that binds to HLA‐G expressed on trophoblasts. ILT‐4 plays a key role in promoting immune tolerance at the maternal‐fetal interface. Recent studies indicate that ILT‐4‐expressing dendritic cells are reduced in the peripheral blood and endometrial biopsies of RPL patients, implicating its dysfunction in RPL pathogenesis [9]. Sirtuin 1 (SIRT1), a class III histone deacetylase, is involved in aging, inflammation, reproduction, and cellular processes such as DNA repair and cell cycle regulation. While SIRT1 is expressed in ovarian follicles and impacts follicular development [10], its role in uterine biology remains unclear, despite evidence linking its dysregulation to reproductive abnormalities [11, 12].

In addition, microRNAs (miRNAs) have emerged as critical modulators in RPL pathogenesis. MiRNAs, the short non‐coding RNAs that regulate gene expression post‐transcriptionally, are detectable in extracellular fluids like plasma and serum [13, 14]. Dysregulated miRNA expression is associated with various pathologies, including gynecological and reproductive disorders such as RPL [15, 16, 17]. Notably, *miR‐223‐3p* has been proposed as a potential biomarker for endometrial receptivity by targeting LIF, suggesting therapeutic strategies to enhance pregnancy outcomes [18].

While LIF, SIRT1, and ILT‐4 have individually been implicated in RPL, their interconnected roles and regulatory mechanisms, particularly involving miRNAs, remain poorly understood. This study aims to address this gap by investigating the effects of LIF on *Sirt1*, *Ilt‐4*, and associated miRNA profiles in a murine model of RPL, providing insights into potential therapeutic targets for pregnancy‐related complications.

## 2 Material and Methods

### 2.1 Recombinant LIF: Production and Functional Evaluation

The construction, expression, and purification of the recombinant LIF (rLIF) protein have been previously detailed [19]. Briefly, rLIF protein was expressed in *E. coli* Origami‐(DE3) using a pColdI vector. The transformation used Terrific Broth (TB) media with 50 μg/mL ampicillin, 15 μg/mL kanamycin, and 12.5 μg/mL tetracycline. A culture was initiated in TB with 1% glucose, antibiotics, and 100 μL of the construct, incubated overnight at 37°C. The next day, the culture was diluted 1:50 in TB, and rLIF expression was induced with IPTG at an optical density of 0.9. After overnight incubation at 15°C, cells were harvested, lysed by sonication, and rLIF was purified using Ni‐NTA chromatography. Bradford's method assessed concentration, and purified fractions were dialyzed overnight. SDS‐PAGE confirmed purification. Functional analysis was performed on TF‐1 cells cultured in RPMI‐1640 with 10% FBS, antibiotics, and GM‐CSF at 37°C for 24 h. Serial dilutions of rLIF (0.625–25 ng/mL) were added and incubated for 96 h. Positive controls received commercial rLIF while negative controls lacked GM‐CSF. MTT solution was then added, and absorbance was measured at 570 nm to determine the proliferation effect of rLIF.

### 2.2 Animal Study

The study was conducted on male BALB/c, DBA/2 J, and female CBA/J mice aged 6–8 weeks, with an average weight of 25 ± 2 g, obtained from the Pasteur Institute of Iran. Mice were housed under standard laboratory conditions, including a 12/12 h light/dark cycle, temperature of 22°C, humidity of 55%, and free access to water and food. The CBA/J mice were paired with DBA/2 J or BALB/c after a 4‐month acclimation period. Experiments were conducted by relevant guidelines, including ARRIVE, and approved by the Shahid Sadoughi University of Medical Sciences Ethics Committee in Yazd, Iran (Ethics Code No: IR.SSU.AEC.1402.015).

### 2.3 Study Design

#### 2.3.1 Development of a Recurrent Miscarriage Mouse Model

A mouse model of recurrent abortion is created by mating CBA/J (F) × DBA/2 J (M), while mating CBA/J (F) × BALB/c (M) leads to a normal pregnancy. Vaginal plaque formation is considered Day 0.5 of pregnancy. Pregnant mice are kept in separate cages for further studies. The pregnancy outcomes of the CBA/J × DBA/2 J model used here, including abortion and resorption rates, implantation sites, and live fetuses, have been previously characterized by our [20], confirming the validity of this model for RPL studies.

#### 2.3.2 Study Groups

**FIGURE 1:**
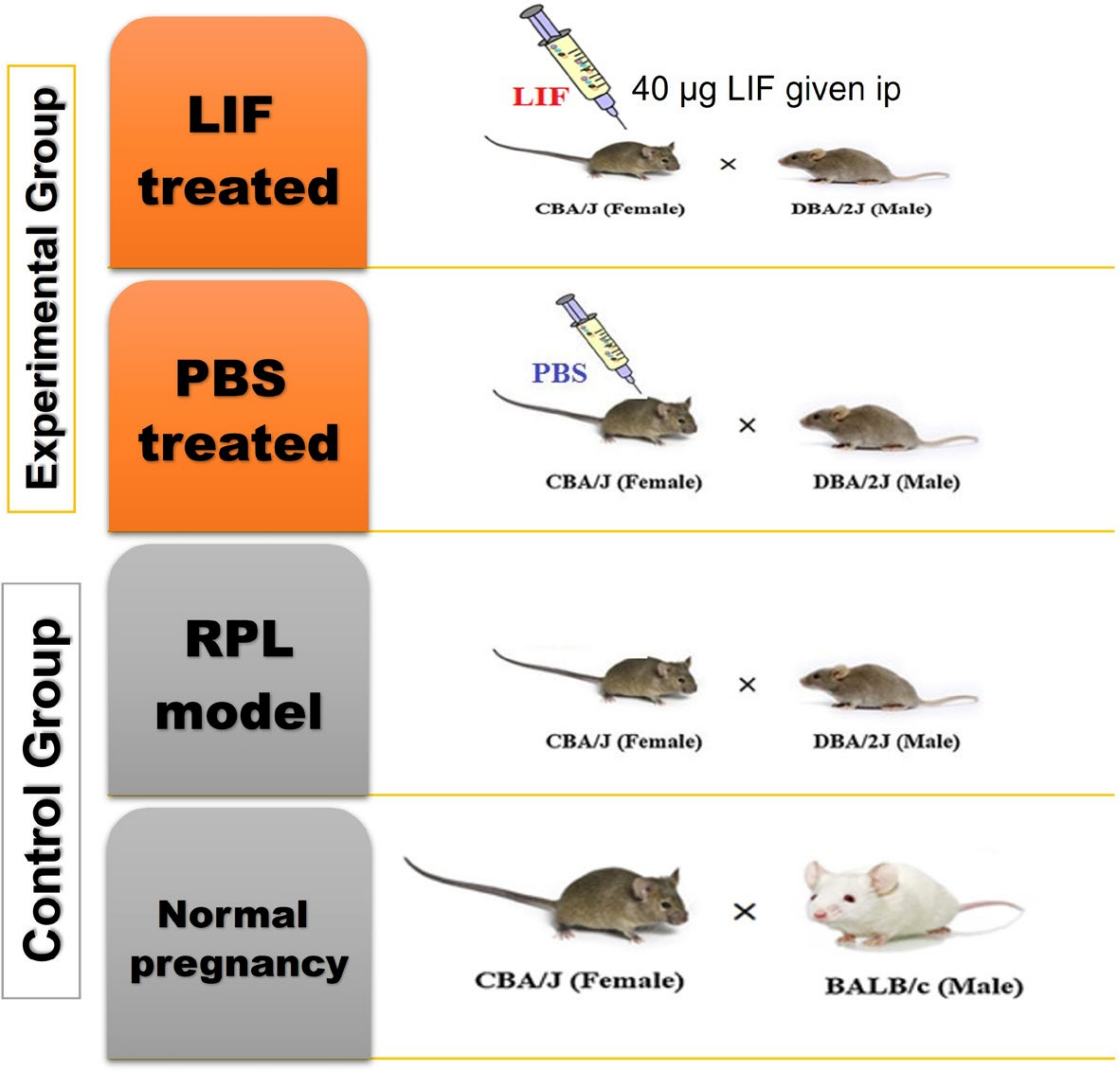
An overview of the studied groups.

In this study, the experimental group included five CBA/J mice mated with DBA/2 J and received 40 μg of LIF intraperitoneally (ip) on Day 3 after vaginal plaque formation. In contrast, the RPL model control group, consisting of five CBA/J mice mated with DBA/2 J, received phosphate‐buffered saline (PBS). Additionally, the normal pregnancy group comprised five CBA/J mice mated with BALB/c [21] (Figure 1). The mice were anesthetized on Days 7 and 14, and their uterine tissue was removed.

#### 2.3.3 mRNA Gene Expression

The uterine tissue RNA was extracted using the RNeasy Mini Kit (Qiagen, USA) according to the manufacturer's instructions. RNA concentration and purity were assessed using a NanoDrop spectrophotometer (Thermo Fisher Scientific, Waltham, MA, USA).

For cDNA synthesis of mRNA targets (*Gapdh*, *Sirt1*, and *Ilt‐4), the* RT‐cDNA Synthesis Kit (Parstous, Iran) was used, following the manufacturer's protocol.

Primers for the target genes (*Gapdh*, *Sirt1*, and *Ilt‐4*) were designed using Primer3 input (version 0.4.0) (Table S1) and their annealing temperatures were optimized. Gene expression analysis was conducted utilizing the SYBR Green methodology usin Ampliqon RealQ Plus 2 × Master Mix (Ampliqon, Denmark) and a Real‐Time PCR system ABI Applied Biosystems (USA). *GAPDH* was used as the internal control for normalization. The relative expression levels of the target mRNAs in all samples were calculated using the 2^−ΔΔCt^ method.

#### 2.3.4 miRNA Quantification

Following total RNA extraction using the RNeasy Mini Kit (Qiagen, USA), miRNA quantification was performed. It is important to note that while the RNA extraction and qPCR amplification steps broadly resembled those for mRNA gene expression, the cDNA synthesis and primer design for miRNAs utilized a specialized commercial kit to ensure specific detection of mature miRNAs. For cDNA synthesis of miRNAs (*miRNA‐199a‐5p*, *miRNA‐138‐5p*, *miRNA‐155‐5p*, and *miRNA‐223‐3p*), we used the BON miR Kit (Bon Yakhteh, Tehran, Iran), following the manufacturer's protocol. This kit employs a proprietary technology that allows for universal reverse transcription of all mature miRNAs in a single reaction, eliminating the need for individual miRNA‐specific reverse primers.

High‐quality, specific primers for the target miRNAs (*miRNA‐199a‐5p*, *miRNA‐138‐5p*, *miRNA‐155‐5p*, and *miRNA‐223‐3p*) and *U6 snRNA* (as an internal control) were designed and synthesized by Bon Yakhteh Company (Tehran, Iran) to be compatible with the BON miR Kit system and SYBR Green methodology. It is important to note that only the forward primer sequences were provided for these commercially supplied miRNA assays.

Real‐time PCR was subsequently conducted on an ABI Real‐Time PCR System (Applied Biosystems, USA), utilizing the Ampliqon RealQ Plus 2 × Master Mix (Ampliqon, Denmark). For normalization, *U6 snRNA* was utilized as the endogenous control, as indicated in the primer's documentation. We confirmed its stable expression across our experimental samples. Relative expression levels of miRNAs were calculated using the 2^−ΔΔCt^ method.

Due to the proprietary nature of the commercial miRNA assay kit (BON miR Kit), full primer sequences and qPCR efficiency data were not disclosed by the manufacturer. Therefore, standard curve‐based efficiency validation could not be independently performed for each miRNA primer pair. Nevertheless, all reactions were conducted strictly according to the manufacturer's optimized protocols for cDNA synthesis and qPCR amplification. Amplification specificity was verified by melt‐curve analysis, and *U6 snRNA* served as a stable internal control, ensuring the robustness and reliability of the quantitative data despite the primer disclosure limitation.

#### 2.3.5 MicroRNA Prediction Method

The *SIRT1* and *ILT‐4* gene symbols were sourced from Gene‐NCBI, while predicted miRNAs were obtained from TargetScan, miRanda, miRmap, miRDB, DIANA TOOLS, and miRWalk [22]. Each database evaluates predicted miRNAs based on specific criteria. The chosen miRNAs were selected according to the following criteria: (1) low context + score and high probability of conserved target, (2) low mirSVR score, (3) high miRmap score, (4) high target score, (5) high miTG score, (6) high miRWalk score, along with being validated in at least five databases. The selected candidate miRNAs were then assessed for mRNA accessibility using Sfold, a statistical tool for RNA secondary structure prediction [23]. miRNAs with a probability greater than 0.5 were selected. Ultimately, after reviewing studies on the roles of the candidate miRNAs, five were chosen for further evaluation in RPL.

### 2.4 Statistical Analysis

mRNA expression levels in all samples were calculated relative to the control gene using the Livak method formulas.

ΔCT=CTof target gene–CTof housekeeping gene.

ΔΔCT=ΔCTof target gene–Average of control groupΔCT.

$$\text{Relative Fold Change}\;\left(\mathrm{RFC}\right)=2^{–\text{ΔΔCT}}$$

The RFC for each sample was calculated using Microsoft Excel. Data analysis was performed with GraphPad Prism (version 10; GraphPad Software, San Diego, CA, USA), utilizing parametric unpaired *t*‐tests or nonparametric tests (Wilcoxon and Mann–Whitney). The Kolmogorov–Smirnov test evaluated data normality, and non‐normally distributed continuous variables were compared using the Mann–Whitney *U* test, with significance set at *p* < 0.05. Results are shown as mean and standard error of the mean (SEM). All graphs and heat map plots were generated with GraphPad Prism, and heat maps were analyzed using the SRplot free online web service (https://www.bioinformatics.com.cn/en) for data analysis and visualization [24].

## 3 Results

### 3.1 Pregnancy Outcomes

**FIGURE 2:**
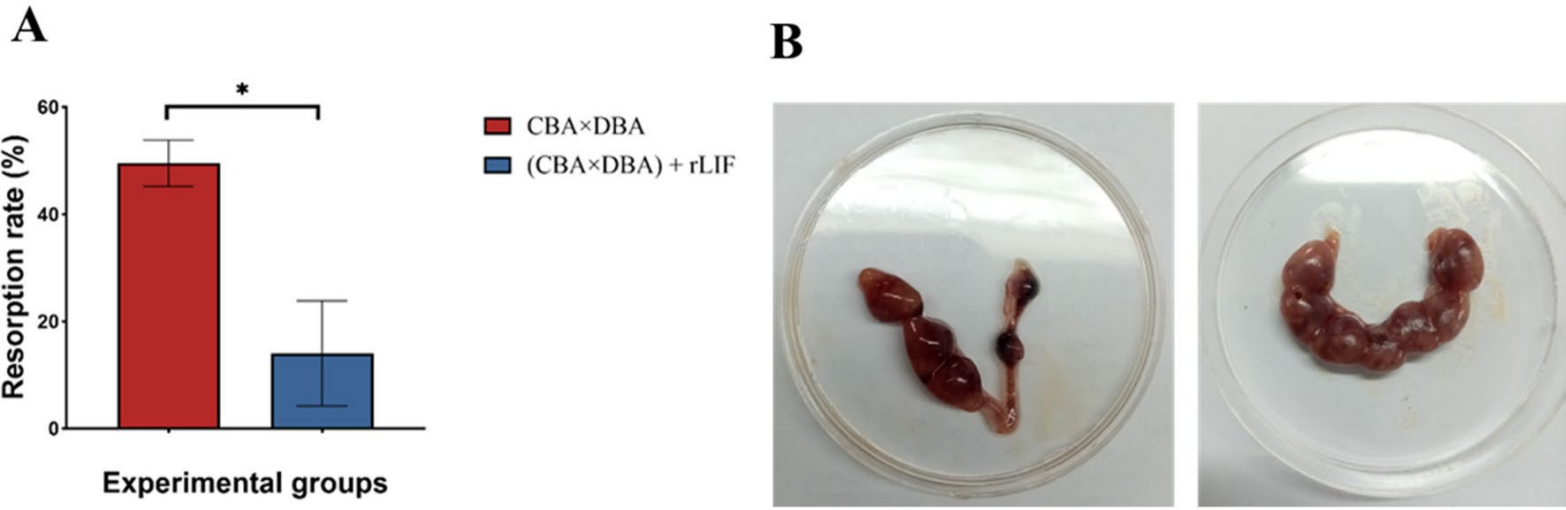
Comparison of resorption rates in the abortion‐prone model. (A) A significant increase in the resorption rate was observed in the CBA×DBA mating group compared to the therapeutic group. Administration of rLIF significantly reduced the resorption rate (*p* = 0.01). Data are presented as Mean ± SEM (*n* = 5 per group) and were analyzed using a Mann–Whitney test. Baseline data adapted from Ansariniya et al. [20] to validate the current experimental model. (B) Representative macroscopic images of uterine horns on Day 14 of gestation. In the control abortion‐prone group (left), resorbed embryos are identified by their markedly smaller size, necrotic appearance, and dark hemorrhagic color compared to viable fetuses. In the rLIF‐treated group (right), a higher frequency of healthy, well‐developed conceptuses is observed, demonstrating the therapeutic potential of rLIF in preventing fetal loss.

Pregnancy outcomes were evaluated to validate the physiological relevance of the abortion‐prone model. The quantitative resorption data presented here were previously reported in our earlier study (Ansariniya et al. [20]) and are included to confirm the validity of the experimental model used for the molecular analyses in the current study. As shown in Figure 2A, the CBA/J × DBA/2 J group exhibited a significantly higher resorption rate compared to the rLIF‐treated group (*p* = 0.01). The mean resorption rate in the abortion‐prone group was approximately 49%, whereas administration of rLIF reduced this rate to approximately 14%.

Resorption rate was calculated as the percentage of resorbed implantation sites relative to the total number of implantation sites per uterus. Consistent with our previous findings, rLIF treatment was associated with reduced embryonic resorption in this model.

Representative macroscopic images of uterine horns at gestational Day 14 are shown in Figure 2B. These images are newly generated for the present study and illustrate the characteristic morphological differences between groups. In the untreated abortion‐prone group, resorbed embryos were identified by their markedly smaller size, necrotic appearance, and dark hemorrhagic coloration compared to viable fetuses. In contrast, the rLIF‐treated group exhibited a higher proportion of morphologically normal and well‐developed conceptuses. No gross morphological differences were observed among viable fetuses between groups.

### 3.2 Gene and miRNA Expression Analysis

**FIGURE 3:**
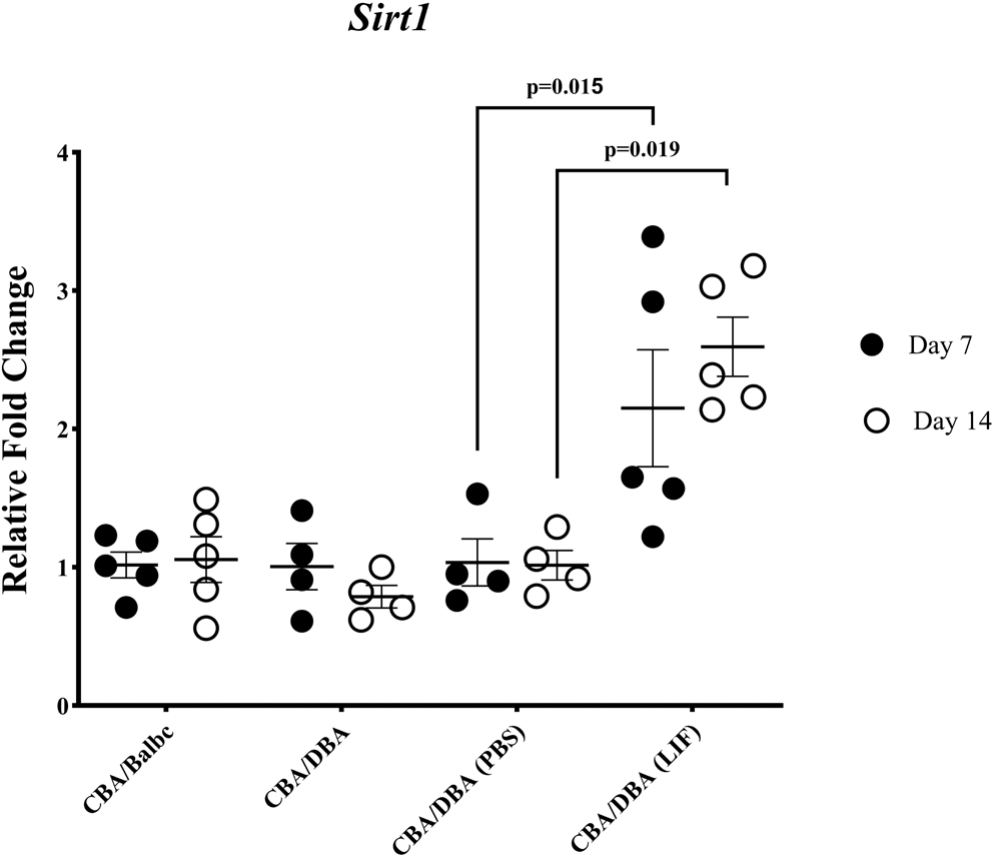
Comparative analysis of *Sirt1* gene expression levels across experimental groups. Individual data points are displayed as scatter plots overlaid with mean ± SEM. Relative fold changes of *Sirt1* expression are shown for the CBA/Balbc, CBA/DBA, CBA/DBA (PBS), and CBA/DBA (LIF) groups on Days 7 (filled circles) and 14 (open circles). Statistical significance is indicated by *p*‐values (*p* = 0.015 and *p* = 0.019), demonstrating significant differences following LIF treatment.

**FIGURE 4:**
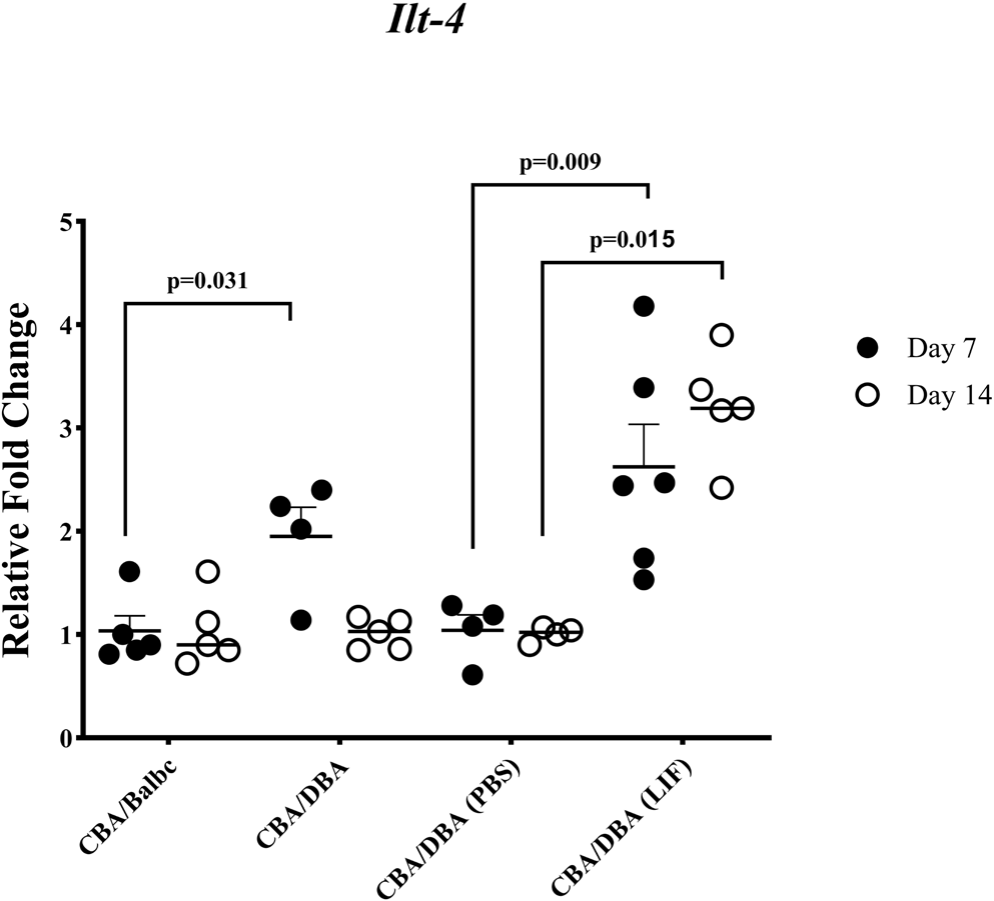
Comparative analysis of *Ilt‐4* gene expression across experimental groups. Scatter plots representing individual data points are shown overlaid with mean ± SEM. Significant differences between groups are indicated by the relevant *p*‐values. Expression levels of *Ilt‐4* exhibit notable alterations among the CBA/Balbc, CBA/DBA, CBA/DBA (PBS), and CBA/DBA (LIF) groups.

The findings of this study revealed a significant upregulation in the expression level of the *Sirt1* gene in the experimental group (CBA/DBA treated with LIF), compared to the RPL model control group (treated with PBS), on Days 7 (*p* = 0.015) and 14 (*p* = 0.019), as illustrated in Figure 3. Similarly, the expression level of the *ILT‐4* gene showed a significant increase in the test group compared to the RPL model control group on Days 7 (*p* = 0.009) and 14 (*p* = 0.015). Furthermore, the untreated CBA/DBA group exhibited a significant increase in *Ilt‐4* expression compared to the normal pregnancy group (CBA/Balbc) on Day 7 (*p* = 0.031), as shown in Figure 4.

**FIGURE 5:**
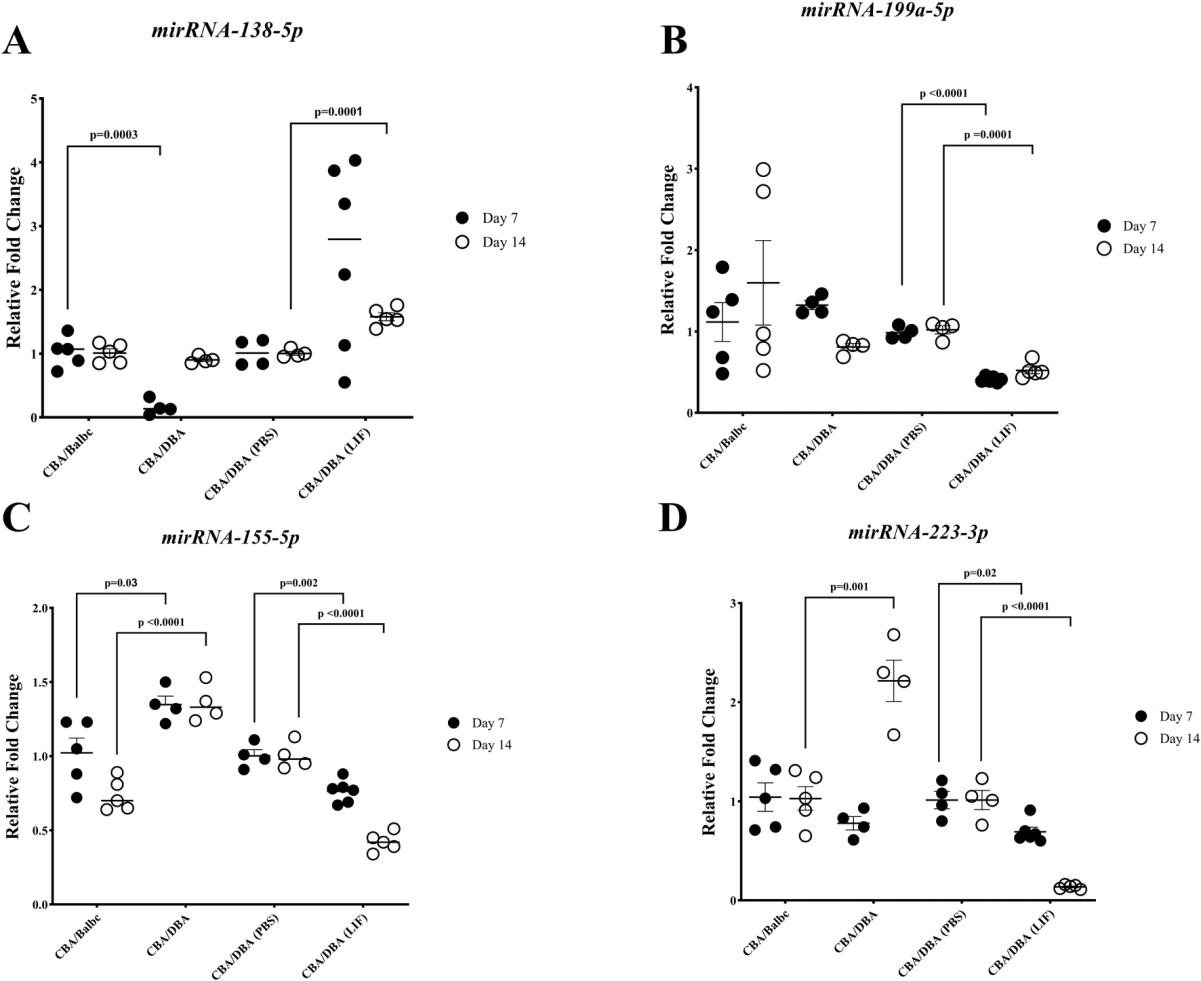
Differential expression of selected microRNAs across experimental groups. Individual data points are presented as scatter plots overlaid with mean ± SEM. Relative fold changes of (A) *miR‐138‐5p*, (B) *miR‐199a‐5p*, (C) *miR‐155‐5p*, and (D) *miR‐223‐3p* are shown for the CBA/Balbc, CBA/DBA, CBA/DBA (PBS), and CBA/DBA (LIF) groups on Days 7 (filled circles) and 14 (open circles). Statistical significance is indicated by the corresponding *p*‐values, demonstrating LIF‐induced modulation of each miRNA and highlighting notable differences between the RPL model and normal pregnancy controls.

The results demonstrated that the expression level of *miR‐138‐5p* was significantly upregulated in the LIF‐treated group compared to the PBS‐treated group on Day 14 (*p* = 0.0001). Moreover, a significant difference was observed between the untreated CBA/DBA group and the normal pregnancy group (CBA/Balbc) (*p* = 0.0003), as illustrated in Figure 5A. As depicted in Figure 5B, the expression level of *miR‐199a‐5p* demonstrated a significant downregulation in the LIF‐treated group on both Day 7 and Day 14 (*p* < 0.0001 and *p* = 0.0001, respectively) compared to the PBS‐treated group. The expression level of *miR‐155‐5p* showed a significant decrease in the LIF‐treated group compared to the PBS‐treated group (*p* < 0.0001). Furthermore, the untreated CBA/DBA group exhibited significant upregulation in *miR‐155‐5p* expression (*p* = 0.03 and *p* = 0.002) compared to the normal pregnancy group (CBA/Balbc), as shown in Figure 5C. Finally, the expression level of *miR‐223‐3p* exhibited a significant downregulation in the LIF‐treated group compared to the PBS‐treated group on Day 7 (*p* = 0.02) and Day 14 (*p* = 0.001), as presented in Figure 5D. Additionally, the untreated CBA/DBA group showed a significant increase in miR‐223‐3p expression compared to the normal pregnancy group (CBA/Balbc) (*p* < 0.0001).

### 3.3 Histopathological Analysis of Placental Tissue

Histopathological examination of placental sections was performed to assess potential structural alterations associated with rLIF treatment. Representative H&E‐stained sections are presented in Figure S1.

In the untreated abortion‐prone group, placental tissues exhibited notable pathological features, including extensive decidua basalis liquefaction and the presence of localized purulent foci, indicative of inflammatory and degenerative processes. In contrast, placentas from the rLIF‐treated group demonstrated preserved structural organization with intact tissue architecture. The severity of decidual liquefaction was markedly reduced, and inflammatory foci were less frequently observed.

Importantly, no treatment‐related structural abnormalities were detected in the labyrinth or spongiotrophoblast layers following rLIF administration. These findings indicate that rLIF treatment does not induce histopathological damage and may contribute to improved placental tissue integrity in the abortion‐prone model.

### 3.4 Correlation Analysis

#### 3.4.1 Correlation Analysis in the LIF‐Treated Group

**FIGURE 6:**
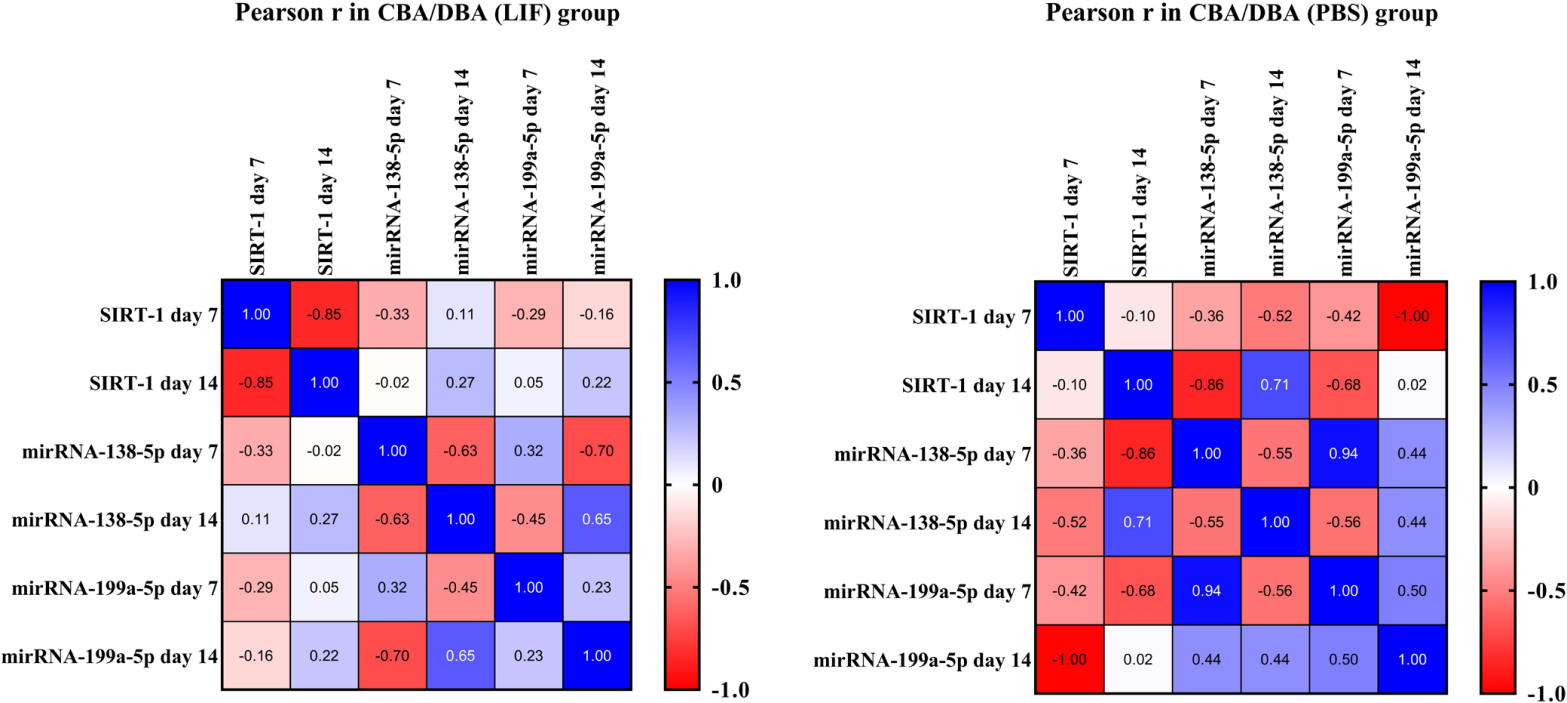
Heatmaps representing the Pearson and the Spearman correlation coefficients between the expression levels of *Sirt1* gene and selected miRNAs (*miR‐138‐5p*, *miR‐155‐5p*, *miR‐199a‐5p*, and *miR‐223‐3p*) at different time points (Day 7 and Day 14) in LIF‐treated and PBS‐treated CBA/DBA groups.

The Pearson correlation analysis in the LIF‐treated group revealed a strong negative correlation between *Sirt1* expression on Day 7 and Day 14 (*r* = −0.8508), indicating an inverse regulatory relationship over time. Additionally, a positive correlation was observed between *miR‐199a‐5p* and *miR‐138‐5p* expression on Day 14 (*r* = 0.65), suggesting a potential cooperative interaction in their regulatory roles. The Spearman correlation analysis in this group identified a strong positive correlation between *miR‐155‐5p* expression on Day 7 and *miR‐223‐3p* expression on Day 7 (*r* = 0.83), and between *miR‐155‐5p* expression on Day 7 and miR‐223‐3p expression on Day 14 (*r* = 0.90). Conversely, a negative correlation was observed between *miR‐155‐5p* expression on Day 14 and *miR‐223‐3p* expression on Day 7 (*r* = −0.80). However, none of these correlations were statistically significant (Figure 6).

#### 3.4.2 Correlation Analysis in the PBS‐Treated Group

The Pearson correlation analysis in the PBS‐treated group revealed minor associations between *Sirt1* expression and the selected miRNAs. *Sirt1* expression on Day 14 demonstrated a strong negative correlation with *miR‐138‐5p* expression on Day 7 (*r* = −0.86). Similarly, *miR‐199a‐5p* expression on Day 7 exhibited a strong positive correlation with *miR‐138‐5p* expression on Day 7 (*r* = 0.94). However, none of these correlations were statistically significant. In contrast, a strong negative correlation was observed between *miR‐199a‐5p* expression on Day 14 and *Sirt1* expression on Day 7 (*r* = −0.996), which was statistically significant (*p* = 0.004) (Figure 6).

**FIGURE 7:**
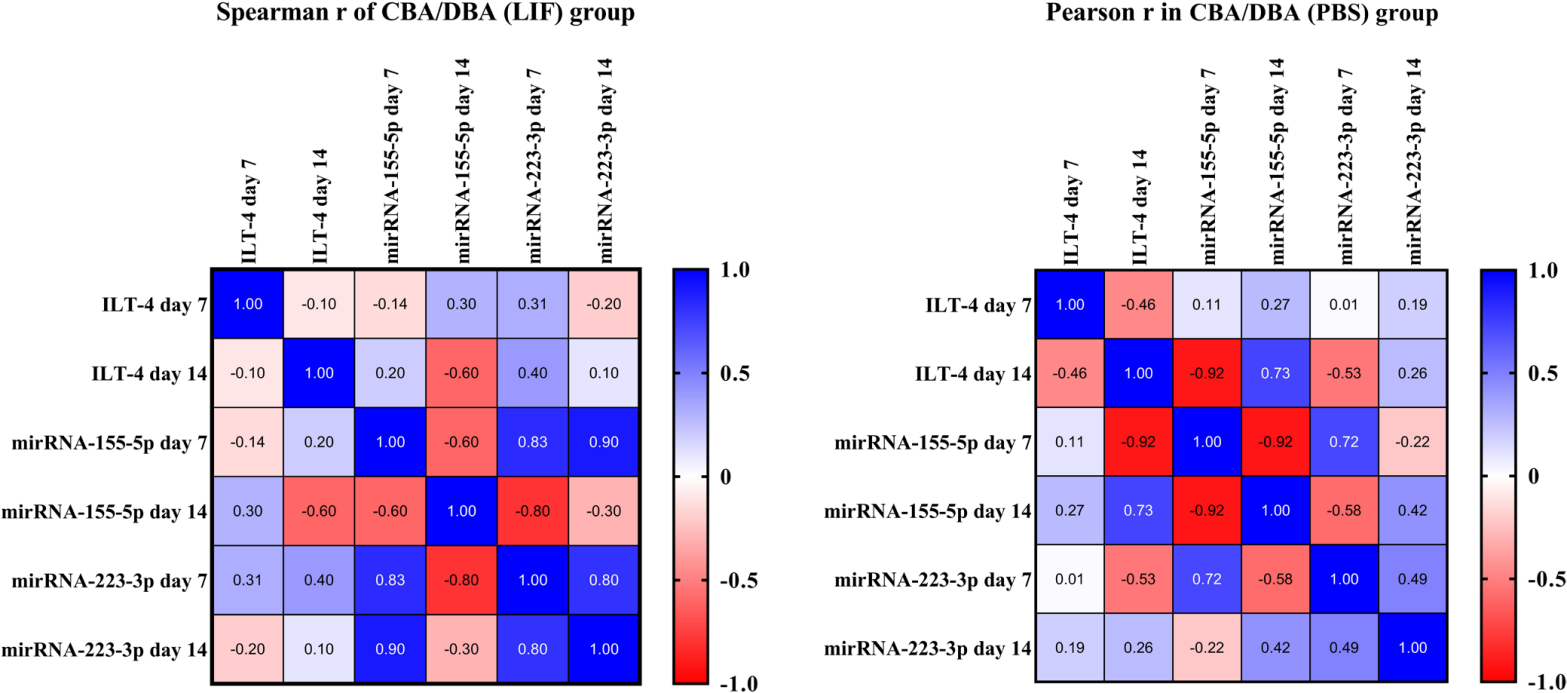
Heatmaps representing the Pearson and the Spearman correlation coefficients between the expression levels of *Ilt‐4* gene and selected miRNAs (*miR‐138‐5p*, *miR‐155‐5p*, *miR‐199a‐5p*, and *miR‐223‐3p*) at different time points (Day 7 and Day 14) in LIF‐treated and PBS‐treated CBA/DBA groups.

For *Ilt‐4* expression on Day 14 exhibited a moderate positive correlation with *miR‐155‐5p* expression on Day 14 (*r* = 0.73), *miR‐155‐5p* expression on Day 7 demonstrated a strong positive correlation with *miR‐223‐3p* expression on Day 7 (*r* = 0.72). *miR‐155‐5p* expression on Day 14 showed a moderate negative correlation with *miR‐223‐3p* expression on Day 7 (*r* = −0.58); also, none of these correlations were statistically significant (Figure 7).

**FIGURE 8:**
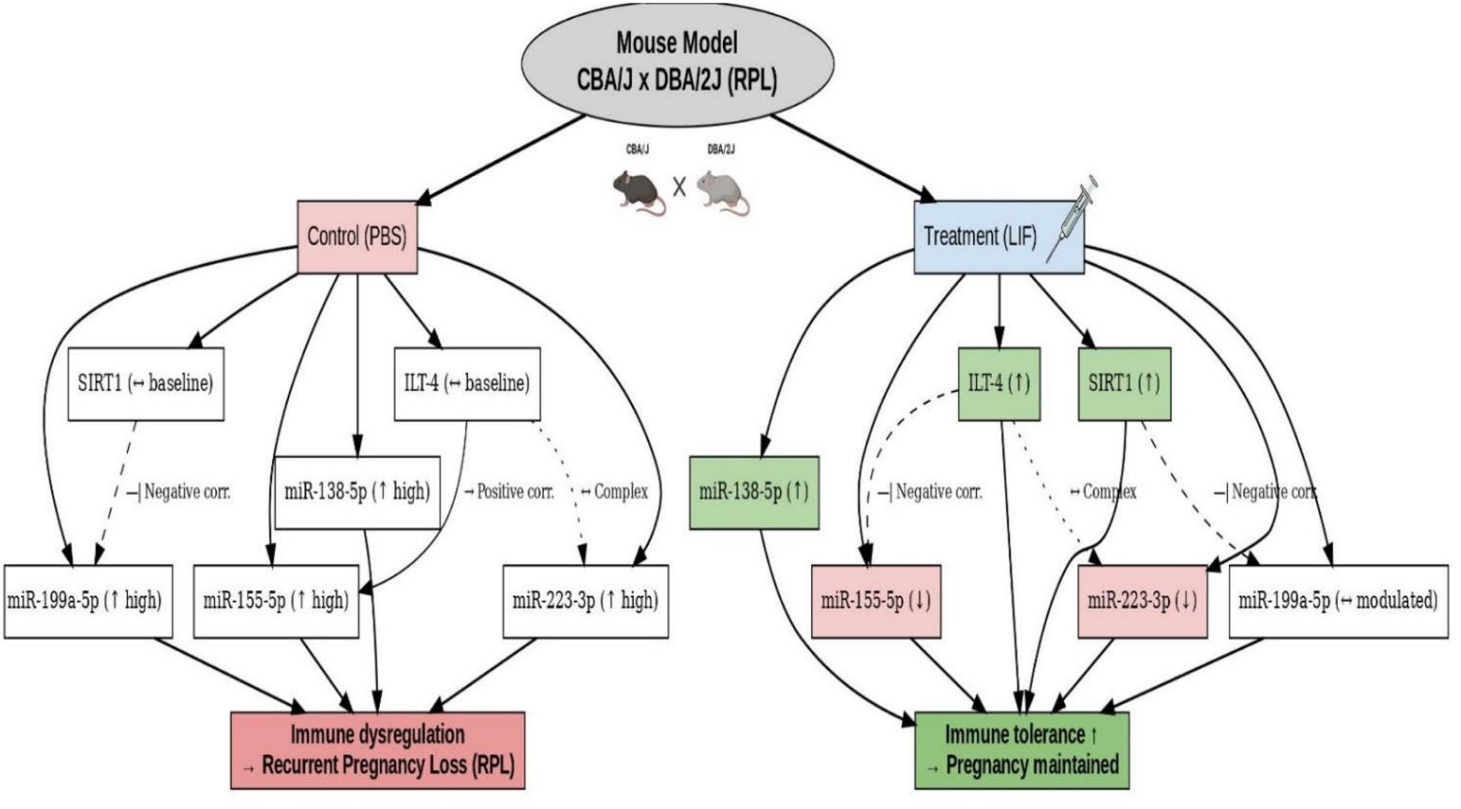
Summarizing the proposed mechanism by which LIF regulates SIRT1/ILT‐4, modulates specific miRNAs, and promotes immune tolerance during early pregnancy.

Figure 8 summarized the proposed mechanism by which LIF regulates *SIRT1/ILT‐4*, modulates specific miRNAs, and promotes immune tolerance during early pregnancy.

## 4 Discussion

RPL, defined as three or more consecutive miscarriages before 20 weeks of gestation, affects 1%–5% of women and remains a significant challenge in reproductive medicine [25]. Genetic abnormalities, including chromosomal disorders, autoimmune diseases, and uterine anomalies, are well‐established causes of RPL. Additionally, recent studies also emphasize the regulatory role of miRNAs in implantation, trophoblast invasion, and endometrial receptivity, processes that are essential for maintaining a healthy pregnancy [26].

This study explored the impact of LIF on the expression of *Sirt1* and *Ilt‐4* genes, as well as the regulation of related miRNAs, in a murine model of RPL. The results demonstrated that LIF administration markedly enhanced the expression of both *Sirt1* and *Ilt‐4* on Days 7 and 14 compared with the PBS‐treated controls. Interestingly, LIF administration markedly enhanced *Sirt1* and *Ilt‐4* expression on Days 7 and 14 compared with PBS‐treated controls. However, no significant differences were observed between the normal pregnancy group (CBA/Balb/c) and the abortion‐prone group (CBA/DBA) at the same time points.

The absence of significant differences in *Sirt1* and *Ilt‐4* expression between normal and abortion‐prone mice at Days 7 and 14 suggests that these genes may be temporally regulated during early gestation. Previous studies using the CBA/J × DBA/2 J model have shown biphasic changes in immune gene expression, particularly around peri‐ and early post‐implantation stages (Days 4–6 and 10–12), which may explain the lack of differences at the sampled time [27]. In addition, compensatory immune mechanisms, such as modulation of uNK cells, macrophage polarization, or cytokine feedback loops, could transiently balance gene expression despite underlying immunological disparities [28]. Hence, future studies including more time points between Days 4 and 12, along with functional immune assessments, are needed to better capture these dynamic regulatory processes.

Taken together, these findings underscore the complexity of immune regulation in RPL and emphasize the need for future longitudinal investigations with larger cohorts and extended time‐point sampling. Such studies will be essential to delineate the dynamic expression profiles of *Sirt1* and *Ilt‐4* and clarify their precise contribution to the pathophysiology of early pregnancy loss.

SIRT1, a NAD^+^‐dependent deacetylase, maintains cellular homeostasis by regulating oxidative stress, inflammation, and immune responses, partly through activating antioxidant transcription factors such as FOXP3 and NRF2 [29, 30]. Heightened oxidative stress, often linked to impaired placental development, leads to complications such as spontaneous abortion [31]. Furthermore, SIRT1 modulates immune tolerance by deacetylating and regulating critical immune mediators. At the maternal‐fetal interface, this modulation ensures a balanced interaction between the maternal immune system and fetal antigens [32]. Evidence indicates that reduced SIRT1 activity is linked to elevated inflammatory markers and impaired angiogenesis in cases of spontaneous abortion, ultimately exacerbating placental insufficiency [33].

The interconnected pathways of LIF and SIRT1 demonstrate their complementary roles in regulating cellular processes. While LIF stimulates cytokine release and immune responses, SIRT1 ensures that these responses do not exacerbate chronic inflammation by suppressing NF‐κB activity. In energy metabolism, LIF indirectly supports AMPK activation through stress‐response signaling, while SIRT1 directly activates AMPK via LKB1 deacetylation [34, 35, 36]. The significant increase in *Sirt1* expression in LIF‐treated mice on Days 7 and 14 suggests that LIF modulates key molecular pathways related to immune regulation and cellular energy balance.

ILT‐4, referred to as Leukocyte Immunoglobulin‐Like Receptor Subfamily B Member 2 (LILRB2), belongs to the family of immunoglobulin‐like receptors (Ig‐like receptors) that are predominantly found on myeloid cells, including monocytes, macrophages, and dendritic cells [37]. ILT‐4, an inhibitory receptor expressed on dendritic cells and macrophages, interacts with HLA‐G on extravillous trophoblasts to promote Treg induction and maintain immune tolerance at the maternal‐fetal interface [38]. This interaction creates an environment conducive to successful implantation and maintenance of pregnancy [39]. However, in pregnancies complicated by conditions such as preeclampsia, the expression of ILT4 and HLA‐G on decidual APCs is significantly reduced. This reduction correlates with defective Treg induction, leading to impaired immune regulation at the maternal‐fetal interface. Such deficiencies are often linked to pregnancy complications, including recurrent miscarriage, preeclampsia, and implantation failure [40].

Although we did not measure baseline uterine LIF in this study, previous work has demonstrated reduced LIF expression in women with unexplained infertility and its essential role in murine implantation [41, 42]. This limitation should be addressed in future studies by directly quantifying endogenous LIF expression in our RPL model to better understand its contribution to the observed molecular and phenotypic changes.

The LIF pathway is known to play a pivotal role in controlling stem cell differentiation and regulating immune responses through the activation of Jak–STAT signaling [43]. This pathway contributes to maintaining immune balance and prevents severe inflammation by inhibiting the overactivation of the immune system [44]. On the other hand, ILT‐4 suppresses activating pathways such as NF‐κB and MAPK, further contributing to the inhibition of excessive immune responses [45]. Both pathways are essential in preventing autoimmune reactions by regulating or suppressing immune responses. In this study, the expression levels of the *Ilt4* gene in a spontaneous abortion mouse model were analyzed. It was observed that untreated mice exhibited a significant increase in *Ilt4* expression on Day 7 of pregnancy. Notably, LIF treatment led to a further significant increase in *Ilt4* expression on Days 7 and 14 compared to PBS treatment. These findings suggest that LIF may play a critical role in enhancing the immune inhibitory environment by upregulating *Ilt4* expression through the activation of Jak–STAT signaling. Overall, these results highlight the interconnected roles of the LIF and ILT4 pathways in pregnancy, demonstrating that LIF not only supports implantation and early pregnancy maintenance but also contributes to the fine‐tuning of immune responses necessary for fetal survival.

In the context of miRNAs, the expression of *miR‐138‐5p* was significantly increased in the LIF‐treated group, while the expression of *miR‐155‐5p*, *miR‐199a‐5p*, and *miR‐223‐3p* was significantly decreased. In the untreated group, the expression of *miR‐223‐3p* was significantly increased compared with the normal pregnancy group. On the other hand, a strong and significant negative correlation was observed between *Sirt1* expression at Day 7 and *miR‐199a‐5p* at Day 14 in the PBS‐controlled group, indicating cross‐regulation of these molecules.

*miR‐138‐5p* inhibits inflammatory pathways such as NF‐κB and MAPK and influences apoptosis at the implantation site [46, 47]. In the current study, while a negative correlation was observed between the expression levels of this miRNA and *Sirt1* in both LIF and PBS treatment groups, the correlation did not reach statistical significance. This lack of significance may be attributed to the limited sample size, which could have reduced the statistical power of the analysis. The study also demonstrated a significant reduction in *miR‐138‐5p* expression in the RPL mouse model compared with the normal group, consistent with previous reports [48]. Notably, LIF administration in the RPL model resulted in a marked increase in the expression of this miRNA, suggesting that LIF may help restore its expression and thereby contribute to the modulation of inflammatory responses and the enhancement of immune tolerance at the maternal‐fetal interface.

Several studies have highlighted the pivotal role of *miR‐155‐5p* as a regulatory factor in recurrent miscarriage. The overexpression of *miR‐155‐5p* disrupts critical processes during early pregnancy by reducing the growth and invasive capabilities of trophoblast cells, thereby impairing placental formation [49]. Furthermore, *miR‐155‐5p* suppresses epithelial‐mesenchymal transition (EMT), a process vital for trophoblast invasion, which directly contributes to defective placental development [50]. In line with these findings, the present study demonstrated that *miR‐155‐5p* expression was significantly elevated in a mouse model of recurrent miscarriage. Interestingly, the study revealed that treatment with LIF modulated this expression, leading to a reduction in *miR‐155‐5p* levels. These results not only corroborate the established role of *miR‐155‐5p* in miscarriage pathology but also suggest a potential therapeutic avenue wherein LIF can mitigate its detrimental effects.

A study has shown that increased expression of *miR‐199a‐5p* may lead to decreased expression of key genes such as LIF. Increased *miR‐199a‐5p* has been observed in patients with RPL, which leads to reduced endometrial receptivity [50]. As a result, the current study found that LIF administration reduced the expression of this miRNA. Also, in a clinical study, peripheral blood mononuclear cells (PBMC) infusion into patients with a history of recurrent implantation failure was able to reduce *miR‐199a‐5p* expression and simultaneously increase *LIF* gene expression, which led to an improvement in the endometrial environment and an increase in implantation success rate [51]. In the setting of recurrent miscarriage, high expression of *miR‐199a‐5p* and decreased *Sirt1* levels may lead to reduced endometrial receptivity, increased inflammation, and impaired pregnancy stability. This negative correlation suggests that targeting *miR‐199a‐5p* may help upregulate *Sirt1* and improve conditions for pregnancy maintenance.

Increased expression of *miR‐223‐3p* can have detrimental effects on embryo implantation. In particular, this miRNA is associated with the suppression of LIF gene expression. Reduced expression of LIF reduces the ability of the blastocyst to attach to the endometrium and the readiness of the endometrial stroma to accept the embryo [52]. Animal studies have shown that increased *miR‐223‐3p* can inhibit pinnapod formation and lead to reduced implantation success and recurrent miscarriages. In one study, it was found that decreased expression of this miRNA during the period of endometrial receptivity increased the expression of *LIF* and other genes associated with endometrial receptivity, thereby increasing the likelihood of successful embryo implantation [50]. This study showed that LIF injection was associated with a significant reduction in *miR‐223‐3p* expression in animal models. This reduction could create a more favorable environment for increased expression of critical genes related to embryo implantation (such as *LIF*), thereby helping to improve implantation and pregnancy maintenance. The reduction in *miR‐223‐3p* expression by LIF may be mediated by increased LIF expression and enhanced effects on pinnapod formation. This process improves embryo adhesion to the endometrium and increases the likelihood of pregnancy success [53].

Although our experiments were performed in a murine model of RPL, the findings may have significant translational relevance. First, the observed upregulation of *Sirt1* and *Ilt‐4* after LIF administration suggests that these pathways could represent therapeutic targets to improve immune tolerance and reduce oxidative stress at the maternal‐fetal interface. Insufficient activity of SIRT1 has already been reported in women with unexplained RPL, correlating with impaired placental angiogenesis and elevated inflammatory mediators [33]. Similarly, decreased frequencies of ILT‐4–expressing dendritic cells have been documented in endometrial biopsies of RPL patients, supporting the clinical relevance of our murine data [9].

Second, the changes we observed in *miR‐155‐5p*, *miR‐199a‐5p*, and *miR‐223‐3p* expression are consistent with studies in human cohorts. For example, overexpression of *miR‐155‐5p* has been linked to impaired trophoblast invasion in women with RPL [49], while elevated *miR‐199a‐5p* is associated with reduced endometrial receptivity [51]. Moreover, altered levels of circulating *miR‐223‐3p* have been reported in women with idiopathic RPL [52]. This overlap suggests that our murine findings could guide the development of non‐invasive biomarkers, such as serum or uterine fluid miRNAs, for early detection of women at risk.

Finally, the demonstration that recombinant LIF administration modulates these pathways highlights its potential as a therapeutic candidate. While recombinant cytokine therapies face challenges regarding dosage and delivery, the observed molecular effects encourage further studies in human trophoblast and endometrial cells, followed by well‐designed clinical trials. Collectively, these data provide a foundation for translating LIF‐based interventions into clinical strategies for RPL management.

## 5 Limitation

This study has several limitations. First, the sample size was relatively small (*n* = 5 per group), reducing statistical power, particularly in correlation analyses. Therefore, interpretations should be made cautiously, and replication in larger cohorts is necessary.

Second, although LIF administration did not produce observable adverse effects in our model, its long‐term fetal safety remains insufficiently characterized. Previous studies using high‐dose LIF (5000 U/mL) during blastocyst transfer reported no developmental abnormalities [54]; however, comprehensive toxicological and long‐term assessments are still required.

Third, our study did not assess immune cell subsets such as uNK cells, Tregs, or macrophage polarization. Integration of flow cytometry or immunohistochemistry in future work will be crucial to understanding the cellular mechanisms connecting LIF with *Sirt1* and *Ilt4* regulation.

Fourth, the study did not measure endogenous uterine *Lif* levels. This important variable should be included in future experiments to clarify the baseline dysregulation of the LIF pathway in RPL.

Furthermore, the lack of data regarding placental weight and fetal growth parameters limits our ability to fully assess the long‐term clinical implications of LIF levels; this warrants further investigation in future studies.

Finally, the study employed a single‐dose regimen of rLIF. Although this approach was intentionally selected to minimize injection‐related stress in the highly sensitive CBA/J × DBA/2 abortion‐prone model and to target the critical pre‐implantation window (gestational Day 3), it limits definitive conclusions regarding dose–response relationships. Moreover, potential interactions between LIF and other immunoregulatory mediators were not systematically explored. Future studies using multiple dosing regimens, varying concentrations, or sustained‐delivery strategies may help define the optimal therapeutic range and further clarify synergistic or context‐dependent immunological effects.

## 6 Conclusion

In conclusion, beyond the mechanistic insights obtained in a murine model, our results align with several clinical observations in women with RPL. The regulatory effects of LIF on *SIRT1*, *ILT‐4*, and specific miRNAs not only improve our understanding of pregnancy maintenance but also open avenues for translation into clinical practice. These molecules may serve as potential biomarkers for patient stratification and as therapeutic targets for novel interventions. Future studies should validate these findings in human tissues and explore clinical applications, including miRNA‐based diagnostics or LIF‐based therapies, to ultimately improve outcomes in women suffering from RPL.

## Conflicts of Interest

The authors declare no conflicts of interest.

## Supporting information


**FIGURE S1:** rmb270062‐sup‐0001‐Supinfo.docx. *Histopathological evaluation of placental tissues*. Representative H&E‐stained placental sections (400× magnification) from experimental groups. (A) Untreated abortion‐prone group showing extensive decidua basalis liquefaction and localized purulent foci, indicative of inflammatory and degenerative changes. (B) rLIF‐treated group demonstrating preserved placental architecture with reduced liquefaction and decreased inflammatory foci. No structural abnormalities were observed in the labyrinth or spongiotrophoblast layers following rLIF treatment.
**TABLE S1:** The sequences of primers.

## Data Availability

Data sharing not applicable to this article as no datasets were generated or analysed during the current study.
