# Supplementary material for: The Impact of Leukemia Inhibitory Factor on Sirtuin1, Ilt‐4, and Related microRNAs Expression in a Mouse Model of Recurrent Pregnancy Loss
Source: Reprod Med Biol. 2026 Jun 18;25(1):e70062. doi: 10.1002/rmb2.70062 (PMC13279882; doi:10.1002/rmb2.70062)
Supplement: Supplementary file 1 — FIGURE S1: rmb270062‐sup‐0001‐Supinfo.docx. Histopathological evaluation of placental tissues. Representative H&E‐stained placental sections (400× magnification) from experimental groups. (A) Untreated abortion‐prone group showing extensive decidua basalis liquefaction and localized purulent foci, indicative of inflammatory and degenerative changes. (B) rLIF‐treated group demonstrating preserved placental architecture with reduced liquefaction and decreased inflammatory foci. No structural abnormalities were observed in the labyrinth or spongiotrophoblast layers following rLIF treatment. TABLE S1: The sequences of primers. [file RMB2-25-e70062-s001.docx]

**
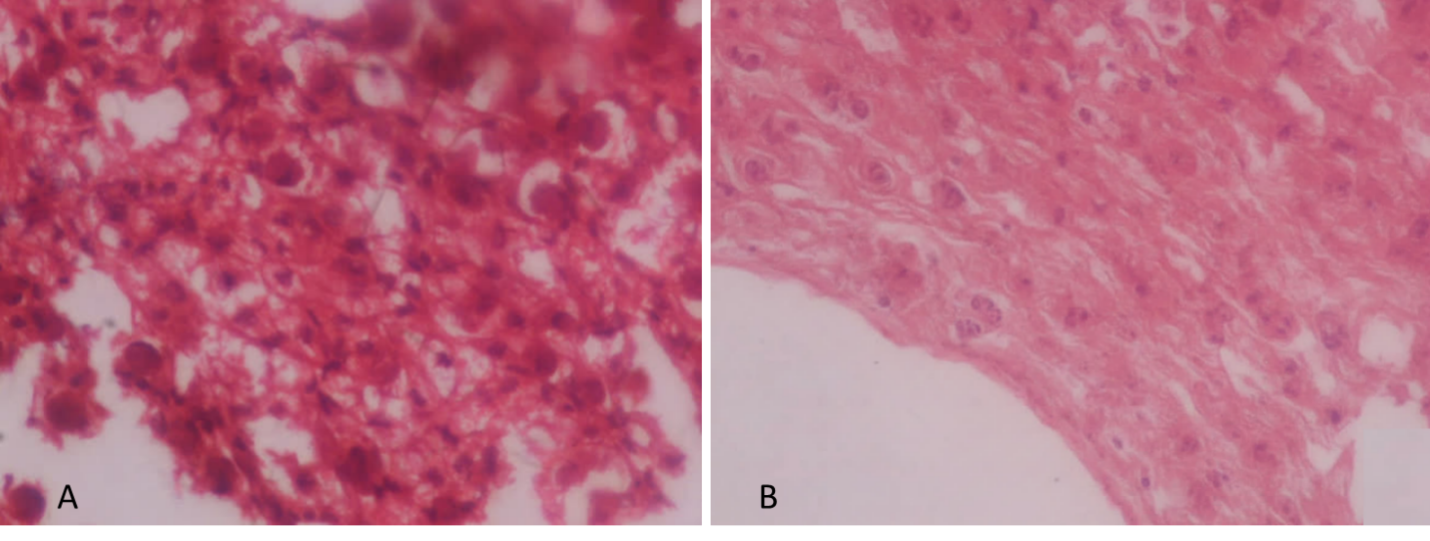
**

**FIGURE S1. Histopathological evaluation of placental tissues.**

Representative H&E-stained placental sections (400× magnification) from experimental groups.

(A) Untreated abortion-prone group showing extensive decidua basalis liquefaction and localized purulent foci, indicative of inflammatory and degenerative changes.

(B) rLIF-treated group demonstrating preserved placental architecture with reduced liquefaction and decreased inflammatory foci. No structural abnormalities were observed in the labyrinth or spongiotrophoblast layers following rLIF treatment.

**TABLE S1** The sequences of primers

| PCR product (bp) | Primer sequence (5'-3') | Accession numbers | | Gene | | Number | |
| --- | --- | --- | --- | --- | --- | --- | --- |
| 197 | F: TGGAGCTGGGGTTTCTGTC R: ACAGAGACGGCTGGAACTG | NM_019812.3 | *Sirt-1* | |  | |  |
| 243 | F: TCTGTGGCCTTCATCCTGTT R: GTTCAGCTCCACTCCATCCT | NM_011095.2 | *Ilt-4* | |  | |  |
| 147 | *F: CACTGCCACCCAGAAGACTG* *R: CCAGTGAGCTTCCCGTTCAG* | NM_001289726.1 | *Gapdh* | |  | |  |
|  | F: TGGCTGTCAGTTTGTCTATA |  | *miR-223-3p* | |  | |  |
|  | F: TAGGCTAAGCGTGATG |  | *miR-155-5p* | |  | |  |
|  | F: GCTGGTGTTGTGAATCA |  | *miR-138-5p* | |  | |  |
|  | F: TTGCCAGTGTTCAGACTA |  | *miR-199-5p* | |  | |  |

F: Forward, R: Reverse
